# Use of the RCOG risk assessment model and biomarkers to evaluate the risk of postpartum venous thromboembolism

**DOI:** 10.1186/s12959-023-00510-6

**Published:** 2023-06-12

**Authors:** Hua Li, Sheng Wan, Jindan Pei, Lu Zhang, Jing Peng, Ronghua Che

**Affiliations:** 1 Department of Obstetrics, Shanghai First Maternity and Infant Hospital, School of Medicine, Tongji University, Shanghai, 200092 China; 2 Shanghai Key Laboratory of Maternal Fetal Medicine, Shanghai Institute of Maternal-Fetal Medicine and Gynecologic Oncology, Shanghai First Maternity and Infant Hospital, School of Medicine, Tongji University, Shanghai, 200092 China

**Keywords:** Postpartum venous thromboembolism (VTE), The RCOG risk assessment model, Biomarkers

## Abstract

**Background:**

Venous thromboembolism (VTE) is a leading cause of morbidity and mortality during pregnancy and the puerperium. The vast majority of VTE occurs after childbirth. China has not yet established standard risk assessment model for postpartum venous thromboembolism (VTE), the Royal College of Obstetricians and Gynecologists (RCOG) risk assessment model (RAM) is commonly used in clinic at present. Herein, we aimed to evaluate the validity of the RCOG RAM in the Chinese population and try to formulate a local risk assessment model by combining with other biomarkers for VTE prophylaxis.

**Methods:**

The retrospective study was conducted from January 2019 to December 2021at Shanghai First Maternity and Infant Hospital which has approximately 30,000 births annually, and the incidence of VTE, differences between RCOG-recommended risk factors, and other biological indicators from medical records were evaluated.

**Results:**

The study included VTE (n = 146) and non-VTE(n = 413) women who examined by imaging for suspicion of postpartum VTE. There was no statistical difference in the incidence rate of postpartum VTE between the low-score group (23.8%) and the high-score group (28%) after stratification by RCOG RAM. However, we found that cesarean section (in the low-score group), white blood cell (WBC) ≥ 8.64*10^9/L (in the high-score group), low-density lipoprotein(LDL) ≥ 2.70 mmol/L, and D-dimer ≥ 3.04 mg/L (in both groups) were highly associated with postpartum VTE. Subsequently, the validity of the RCOG RAM combined with biomarkers as a model for the risk assessment of VTE was estimated and the results showed that this model has good accuracy, sensitivity, and specificity.

**Conclusions:**

Our study indicated that the RCOG RAM was not the best strategy for predicting postpartum VTE. Combined with some biomarkers (including the value of LDL and D-Dimer, and WBC count), the RCOG RAM is more efficient when identifying high-risk groups of postpartum VTE in the Chinese population.

**Trial registration:**

This purely observational study does not require registration based on ICMJE guidelines.

**Supplementary Information:**

The online version contains supplementary material available at 10.1186/s12959-023-00510-6.

## Introduction

Venous thromboembolism (VTE), including deep venous thrombosis (DVT) and pulmonary embolism (PE), is a leading cause of morbidity and mortality during pregnancy and the puerperium [1–3]. During pregnancy, the risk of VTE is increased about 5-fold compared to non-pregnant women and becomes 30 to 60-fold at postpartum (especially in 6 weeks post-delivery) [4–7]. The effective prevention and management of VTE and its complications are crucial.

Pregnancy is an acquired and independent risk factor for the development of VTE. The physiological changes in pregnancy alter the balance of hemostasis to favor coagulation and so reduce blood loss during childbirth, such as an increase in the coagulation factor, hypercoagulability caused by the hemostatic system, changes in venous outflow induced by hormones, mechanical obstruction by the uterus, and vascular injury [8–10]. Besides, previous studies have identified some specific characteristics which may increase the risk of postpartum VTE, including obesity, preterm delivery, mode of delivery, previous VTE, postpartum infection, and varicose veins [7, 11–13]. Detailed assessment of pregnant women can establish a risk profile that would guide clinical decisions, and balance potential therapeutic benefits with side effects. But the assessment of VTE risk factors varies depending on the population studied and the data source used.

To reduce pregnancy-related VTE, Risk assessment models (RAMs) have been developed to identify high-risk groups, and to provide early preventive treatment. However, standardized interventions have not been agreed on and recommendations for postpartum thromboprophylaxis vary among international guidelines [5, 14, 15]. It is also unclear which VTE RAM is best to guide decision-making for thromboprophylaxis in clinic and thereby optimize patient care [16]. Since China has not established its own pregnancy-related VTE risk assessment guidelines, the Royal College of Obstetricians and Gynecologists (RCOG) risk assessment model (RAM) is commonly used in China (including our hospital) for the risk score and risk stratification of postpartum VTE at present. whether this model is appropriate for the Chinese postpartum population remains poorly understood. Considering the above, there is an urgent need to understand the risk factors and establish an effective risk assessment model for postpartum VTE in China.

This study aimed to evaluate the effectiveness of the RCOG RAM, discover other biomarkers for postpartum VTE, and formulate a local risk model for VTE prophylaxis that would help to identify postpartum women at high risk of developing VTE who could receive thromboprophylaxis in the Chinese population.

## Materials and methods

### Study population

We conducted a retrospective study from January 2019 to December 2021 in Shanghai First Maternity and Infant Hospital, Tongji University School of Medicine, a specialized hospital of obstetrics and gynecology in Shanghai, which has approximately 30,000 births annually. The data from women examined by imaging for suspicion of pregnancy-related VTE were collected. Inclusion criteria included women who gave birth beyond 28 weeks of gestation and completed regular antenatal and postnatal examinations as well as diagnostic examinations for VTE. Women who used anticoagulant or anti-platelet drugs before delivery, with multiple gestations, with antepartum VTE, or with incomplete clinical data were excluded from this study.

Women diagnosed with VTE during the postpartum period constituted the VTE group. Women confirmed without VTE were selected as the control group (non-VTE group) on the same day.

### Data collection

Data were abstracted from the complete electronic medical chart (EMR), including sociodemographic characteristics, reproductive history, gynecologic history, previous contraceptive use, obstetric characteristics, and the results of biomarkers and imaging examinations. At 42 days postpartum, all women came to the hospital for regular examination and these data were also collected.

The data of biomarkers containing D-dimer level, white blood cell counts, platelet counts, fibrinogen level, brain natriuretic peptide (BNP), and other plasma lipid levels were collected. Blood samples for testing D-dimer were collected on postpartum day 2, and samples for other biomarkers were collected on postpartum day 1. All hematology samples were sent immediately to the laboratory for testing after collection. Plasma concentrations of D-dimer was measured by using an immunoturbidimetric Innovance D-Dimer Assay (Siemens Healthcare Diagnostics, Marburg, Germany) and a Sysmex CN-6000 instrument (TOA Medical electronics Co., Kobe, Japan). All biomarker values were obtained from the same laboratory affiliated with the hospital.

### Assessment risk of postpartum VTE

Modified 2015 RCOG Guideline on pregnancy-related VTE was used to determine the postpartum VTE risk scores and risk levels among postpartum women [5]. The risk of postpartum VTE was divided into 2 levels: low score (< 2 points and without high-risk factors, no need for thromboprophylaxis), high score (≥ 2 points and/or with high-risk factors, need for thromboprophylaxis). If the risk score greater than or equal to 2 was recommended for thromboprophylaxis including the intermediate and high-risk group in the RCOG RAM, so we classified those as a high-score group. All scores were calculated carefully by obstetricians promptly on day 1 postpartum. The detailed scoring method for VTE was shown in Supplementary Table 1.

### The diagnosis of VTE

Imaging evidence was confirmed as the diagnostic criteria for VTE [17]. Deep venous thrombosis (DVT) was diagnosed by lower extremity venous color Doppler ultrasound, and pulmonary embolism (PE) was diagnosed by computer tomography pulmonary angiography (CTPA).

Imaging examinations were required for suspicion of pregnancy-associated VTE if the following conditions were present [18]: (1) with suspicious symptoms of VTE, including pain or tenderness when moving limbs, swelling of the limbs, measurement of inconsistencies in the circumference of the bilateral limbs, or unexplained dyspnea, chest pain or cough; or (2) with multiple high-risk factors and the high D-dimer level, the clinician considered that the probability for VTE was great. Anticoagulation and antithrombotic therapy would be applied immediately when imaging examination confirmed the diagnosis of VTE.

### Statistical analyses

The statistical analysis was performed by SPSS 22.0 (IBM Corporation, New York, USA). Quantitative data were described as means ± standard deviation (SD). In univariate analysis, continuous variables were analyzed using the t-test or the Mann-Whitney U test, and the categorical variables were analyzed using the Chi-square test, Yate’s correction of continuity, or Fisher’s exact test. The continuous variables with statistical significance in biomarkers were analyzed by the receiver operating characteristic curve (ROC). According to their cut-off values, they were converted to binary classification variables. Before and After stratification by RCOG risk scores, the high-risk factors and other biomarkers were compared among the no-VTE group and the VTE group. Furthermore, to estimate the risk factors of VTE, multiple logistic regression was performed. Based on the logistic regression model, the predicted probability and area under the curve (AUC) of VTE were calculated. Statistical significance was set at p < 0.05.

A checklist of TRIPOD was shown as Supplementary Tables 2 to identify necessary items regarding the reporting of the forecasting model.

## Results

### Comparison of the general characteristics

**Fig. 1:**
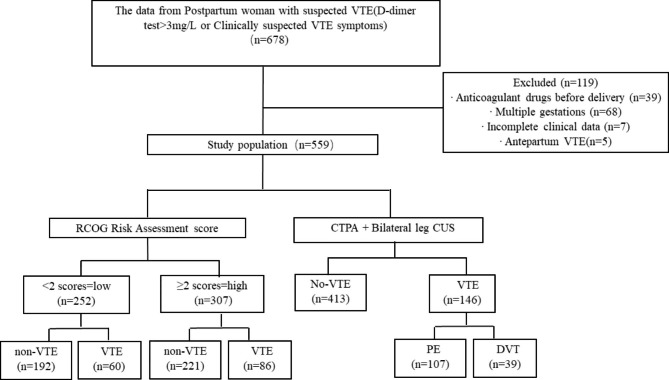
Study population (Flow chart)

We collected data from 678 pregnant women after screening and 559 women who fulfilled the criteria were enrolled in this study. In our cohort, 146 women were divided into the VTE group, including 107 PE events and 39 DVT events; 413 women without VTE were assigned to the non-VTE group. According to RCOG RAM, 252 women with a low-risk score (low-score group) and 307 women with a high-risk score (high-score group) were finally included (Fig. 1).

The general characteristics of the patients in the different divided groups were compared. As shown in Table 1, Age, gravidity, pre-delivery weight and BMI, pre-pregnancy weight and BMI, neonatal birth weight, and gestational age at delivery in the high-score group were significantly higher than those of the low-score group (P < 0.05). Only neonatal birth weight and gestational age at delivery were the statistical difference between VTE and non-VTE groups (P < 0.05).

**Table 1:** Comparison of general characteristics between different divided groups

Variable	low- score group	high- score group		*P*		non-VTE group	VTE group	*P*	
	(n = 252)	(n = 307)				(n = 413)	(n = 146)		
age (years)	30.74 ± 3.22		33.43 ± 4.26		0.000	32.11 ± 4.03	32.53 ± 4.09	0.281	
Gravidity (n)	1.61 ± 0.86		1.99 ± 1.30		0.000	1.79 ± 1.13	1.90 ± 1.15	0.284	
Parity (n)	1.19 ± 0.41		1.25 ± 0.49		0.097	1.21 ± 0.45	1.28 ± 0.48	0.101	
Weight before pregnancy (kg)	55.55 ± 8.04		58.38 ± 9.71		0.000	57.17 ± 9.09	56.94 ± 9.16	0.792	
Weight before delivery (kg)	69.18 ± 9.07		72.18 ± 10.41		0.000	71.04 ± 9.94	70.23 ± 9.95	0.400	
Gain weight during pregnancy (kg)	13.62 ± 4.82		13.80 ± 4.78		0.670	13.87 ± 4.66	13.29 ± 5.38		0.252
BMI before pregnancy (kg/m^2^)	21.22 ± 2.71		22.50 ± 3.48		0.000	22.00 ± 3.19	21.74 ± 3.32		0.403
BMI before delivery (kg/m^2^)	26.43 ± 2.98		27.81 ± 3.69		0.000	27.32 ± 3.39	26.82 ± 3.63		0.129
Neonatal birth weight(g)	3388.06 ± 427.41		3249.46 ± 629.66		0.002	3345.09 ± 525.30	3218.18 ± 612.55		0.017
Gestational age at delivery (weeks)	39.17 ± 1.30		38.31 ± 2.33		0.000	38.81 ± 1.85	38.39 ± 2.27		0.029

Abbreviation:

*BMI* Body mass index

### Risk scoring and risk stratification based on the RCOG RAM

Table 2 showed that the mean RCOG score of women in the VTE group was much higher than that of the non-VTE group (1.90 ± 1.24 vs. 1.65 ± 1.23, P = 0.035). However, VTE was diagnosed in 86(28%)of 307 patients in the high-score group and 60 (23.8%)of 252 patients in the low-score group. The incidence rate of VTE between the two groups was very close and there was no statistical difference(P = 0.26).

**Table 2:** The RCOG risk score and risk level in the VTE group and non-VTE group

	non-VTE group (n = 413)	VTE group (n = 146)	P
Score (mean ± SD)	1.65 ± 1.23	1.90 ± 1.24	0.035
Risk level, n (%)			
Low score	192 (76.2)	60 (23.8)	0.260
high score	221 (72.0)	86 (28.0)	

Abbreviations: RCOG The Royal College of Obstetricians and Gynecologists, VTE Venous thromboembolism

### Comparison of the other biomarkers in divided group

Table 3 compares the mean biomarker levels between the VTE Group and the non-VTE group, after stratification by RCOG risk scores. For women with a low score, the mean TC levels in the VTE group were much higher than that of the non-VTE group (6.74±1.36 vs 6.21±1.42, P=0.012), other biomarkers showed no significant differences between the 2 groups (P >0.05). For women with a high score, the mean levels of D-Dimer, LDL, and WBC count were significantly higher in the VTE group than those in the non-VTE group (P=0.049, 0.010, 0.003, respectively). There was no statistical difference in other biomarker levels between VTE and non-VTE groups (P >0.05).

**Table 3:** Comparison of the biomarker Levels between the VTE Group and the no-VTE group, after stratification by RCOG risk scores

Variable	low-risk group				high-risk group		
	non-VTE(n=192)		VTE(n=60)	*P*	non-VTE(n=221)	VTE(n=86)	*P*
D-Dimer(mg/L)	7.10±5.81	7.30±5.23		0.823	5.36±5.33	6.77±6.05	**0.049**
Fibrinogen (g/L)	3.63±0.74	3.66±0.80		0.752	3.85±0.87	3.77±0.90	0.492
BNP(pg/ml)	66.22±64.05	77.60±78.93		0.260	80.37±82.64	99.52±100.16	0.088
Hcy (umol/L)	6.46±1.84	6.41±1.89		0.861	7.31±4.86	7.04±3.05	0.668
TG (mmol/L)	3.53±1.90	3.64±1.69		0.689	3.86±1.98	3.94±1.31	0.643
TC (mmol/L)	6.21±1.42	6.74±1.36		**0.012**	6.12±1.36	6.37±1.27	0.144
HDL (mmol/L)	2.08±0.46	1.95±0.43		0.067	1.97±0.47	1.86±0.45	0.105
LDL (mmol/L)	3.48±1.07	3.69±1.08		0.221	3.21±0.90	3.54±0.92	**0.010**
ApoA1(g/L)	1.89±0.38	1.92±0.40		0.566	1.81±0.38	1.80±0.45	0.833
ApoB(g/L)	1.28±0.32	1.34±0.26		0.182	1.29±0.29	1.32±0.32	0.468
WBC(^10^9^/L)	8.36±2.23	8.77±2.18		0.209	8.25±2.11	9.13±2.34	**0.003**
HCT(%)	35.75±2.81	35.69±6.37		0.950	36.13±8.38	35.30±3.42	0.372
Platelet count (10^9/L)	193.28±53.23	180.55±49.74		0.102	192.07±56.45	197.61±51.47	0.430

Abbreviations: *BNP* Brain natriuretic peptide, *Hcy* Homocysteine, *TG* Total glyceride, *TC* Total cholesterol, *HDL* High-density lipoprotein, *LDL* Low-density lipoprotein, *ApoA1* Apolipoprotein *A1, ApoB* Apolipoprotein B, *WBC* White blood cell, *HCT* Red blood cell specific volume

The biomarkers with statistical significance were analyzed by the ROC. As shown in Table 4, the area under the ROC curve (AUC) for the TC, LDL, D-Dimer, and WBC count were 0.577, 0.576, 0.550, 0.585, The ROC curve analysis showed the best cut-off point for TC, LDL, D-dimer level and WBC count within postpartum 48 h was 6.16 mmol/L, 2.70 mmol/L, 3.04 mg/L, 8.64*10^9/L. The p-values in Table 4 were obtained using Delong test. According to the cut-off values, they were converted to binary classification variables.

**Table 4:** ROC Analysis for biomarkers predicting VTE

Variable	AUC	95%CI		P	Cut-off	Sensitivity	Specificity
TC(mmol/L)	0.577	0.518–0.635		0.012	6.16	0.682	0.468
LDL(mmol/L)	0.576	0.519–0.634		0.012	2.70	0.864	0.289
D-Dimer (mg/l )	0.550	0.098–0.492		0.098	3.04	0.917	0.225
WBC(10^^9^/L)	0.585	0.527–0.643		0.005	8.46	0.553	0.592

Abbreviations: *TC* Total cholesterol, *LDL* Low density lipoprotein, *WBC* White blood cell

### Comparison of the risk factors between the VTE and non-VTE group, after stratification by RCOG risk scores

After stratifying the population based on the RCOG risk scores, the risk factors were compared between the 2 groups (Table 5). For women with a lows core, Cesarean section, TC≥6.16 mmol/L, LDL≥2.70 mmol/L, D-dimer≥3.04 mg/L, and WBC≥8.64*10^9/L were significantly associated with postpartum VTE (P<0.05). For women with a high score, we found that the proportions of women in the VTE group with TC≥6.16 mmol/L (P=0.004), LDL≥2.70 mmol/L (P=0.000), D-dimer≥3.04 mg/L (P=0.003), and WBC≥8.64*10^9/L (P=0.004) were higher than those in the non- VTE group. Other risk factors showed no significant differences between the 2 groups.

**Table 5:** Risk factors for postpartum VTE after stratification by RCOG risk scores

	low- score					high- score			
Variable	non-VTE(n=192) N(%)	VTE (n=60) N(%)	X^2^	*P*	non-VTE(n=221) N(%)		VTE(n=86) N(%)	X^2^	*P*
Age≥35 years	21(10.9)	4(6.7)	0.516	0.472	76(34.4)		36(41.9)	1.491	0.222
Parity≥3	1(0.5)	0(0)	/	1.000	5(2.3)		0(0)	/	0.327
BMI before pregnancy(≥30 kg/m^2^)	1(0.5)	0(0)	/	1.000	7(3.2)		2(2.3)	0.000	0.987
BMI before delivery(≥30 kg/m^2^)	31(16.1)	10(16.7)	0.009	0.924	58(26.2)		19(22.1)	0.568	0.451
Medical comorbidities	6(3.1)	0(0)	0.812	0.368	4(1.8)		3(3.5)	0.211	0.646
Cesarean section	62(32.3)	37(61.7)	16.538	**0.000**	210(95)		84(97.7)	0.519	0.417
Pre-eclampsia /eclampsia	10(5.2)	4(6.7)	0.012	0.914	48(21.7)		16(18.6)	0.364	0.546
DM/GDM	16(8.3)	5(8.3)	0.000	1.000	30(13.6)		12(14)	0.008	0.931
Preterm birth	5(2.6)	1(1.7)	0.000	1.000	35(15.8)		20(23.3)	2.317	0.128
IVF	6(3.1)	0(0)	/	0.341	46(20.8)		12(14)	1.902	0.168
PPH (>1 L)	6(3.1)	5(8.3)	2.971	0.085	17(7.7)		7(8.1)	0.017	0.896
Immobility	0(0)	1(1.7)	2.883	0.090	4(1.8)		5(5.8)	2.223	0.136
FGR	0(0)	1(1.7)	/	0.238	5(2.3)		5(5.8)	2.478	0.115
Partum infection	10(5.2)	1(1.7)	0.656	0.418	28(12.7)		12(14)	0.090	0.764
transfusion	5(2.6)	4(6.7)	1.170	0.279	12(5.4)		6(7)	0.268	0.604
TC≥6.16 mmol/L	111(57.8)	44(73.3)	4.651	0.031	104(47.1)		56(65.1)	8.089	**0.004**
LDL≥2.70 mmol/L	111(57.8)	52(86.7)	16.661	**0.000**	96(43.4)		63(73.3)	22.044	**0.000**
D-Dimer ≥3.04 mg/L	166(86.5)	58(96.7)	4.823	**0.028**	154(69.7)		74(86)	8.674	**0.003**
WBC≥8.64*10^^9^	87(45.3)	32(53.3)	1.180	0.277	88(39.8)		50(58.1)	8.397	**0.004**

Abbreviations: *BMI* Body mass index, *DM/GDM* Diabetes mellitus/Gestational diabetes mellitus, *TC* Total cholesterol; *IVF* In-vitro fertilization, *LDL* Low-density lipoprotein, *WBC* white blood cell

### Multivariable analysis

**Fig. 2:**
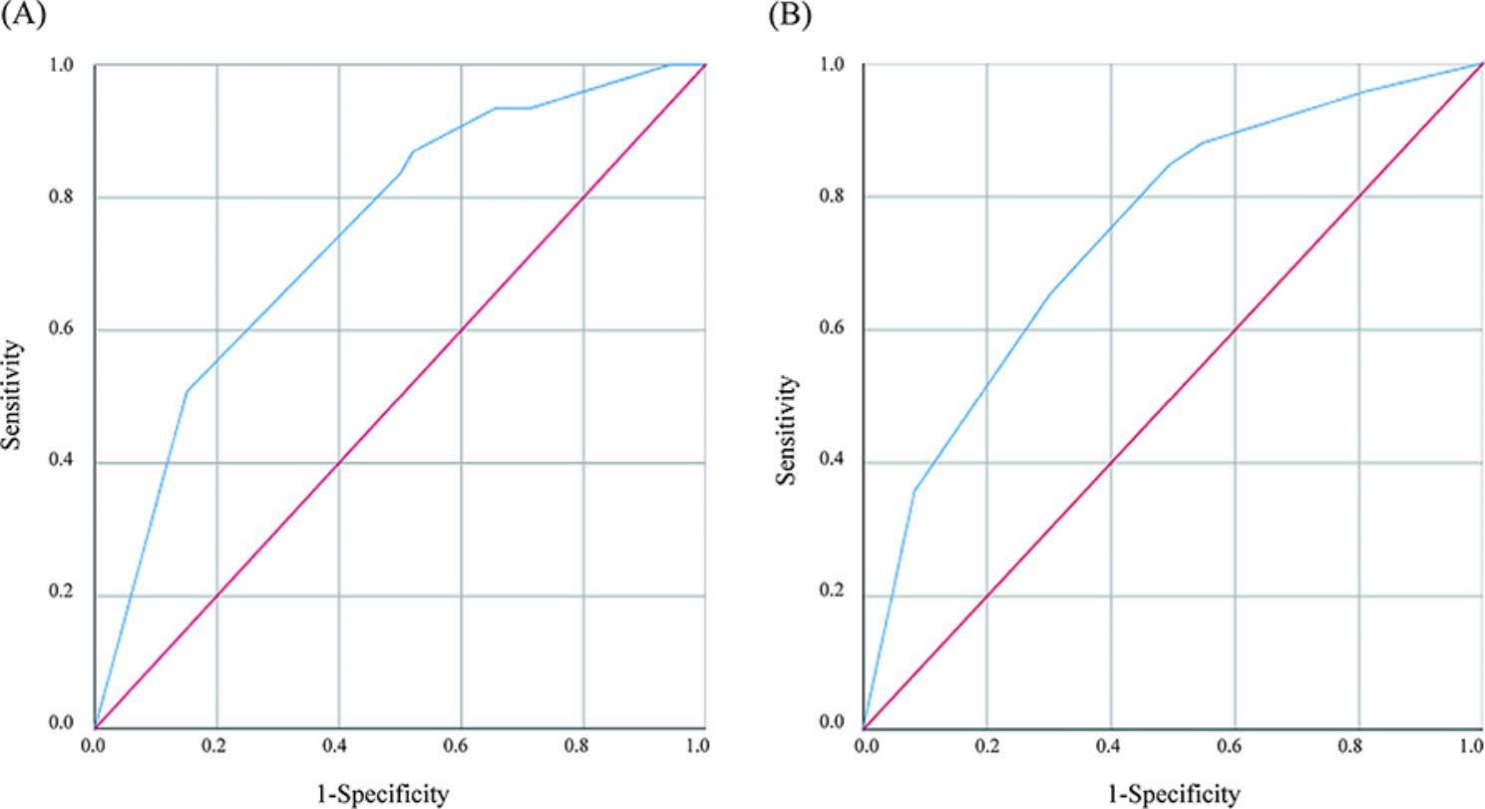
Mean receiver operating characteristic curves of (**A**) low-score group and (**B**) high-score group in prediction model by ROC analysis

Risk factors found to be significantly different in univariate analysis were further entered into a multivariable logistic regression analysis. Before stratification by RCOG risk scores, the cesarean section (adjusted OR = 3.392, 95% CI: 2.041–5.635), LDL (adjusted OR = 3.842, 95% CI: 2.353–6.273), D-dimer (adjusted OR = 2.969, 95% CI: 1.570–5.614), and WBC (adjusted OR = 2.132, 95% CI: 1.399–3.250) were related with postpartum VTE (Table 6). As shown in Table 7, in the low-score group, the results indicated that cesarean section (adjusted OR = 3.399, 95% CI: 1.81–6.38), LDL ≥ 2.70 mmol/L (adjusted OR = 5.16, 95% CI: 2.27–11.74), D-dimer ≥ 3.04 mg/L (adjusted OR = 4.69, 95% CI: 1.03–21.38) were associated with elevated risks of postpartum VTE. In the high-score group, LDL ≥ 2.70 mmol/L (adjusted OR = 4.19, 95% CI: 2.43–7.25), D-dimer ≥ 3.04 mg/L (adjusted OR = 3.05, 95% CI: 1.51–6.16) and WBC ≥ 8.64*10^9/L (adjusted OR = 2.82, 95% CI: 1.67–4.77) were associated with postpartum VTE. Then, based on the obtained logistic regression model, the predicted probability of VTE was calculated. The result (Fig. 2) showed that the AUC was 0.745, the sensitivity was 50.8%, and the specificity was 84.9% in the low-score group; In the high-score group, the AUC was 0.743, the sensitivity was 84.8%, and the specificity was 50.6%. This indicated the Logistic regression model had good discriminative validity, and this model could effectively distinguish the high-risk group of VTE.

**Table 6:** Multivariable Analysis before stratification by RCOG risk scores

Variable	*B*	Wald	P	OR	95% CI
Mode of delivery	1.221	22.232	0.000	3.392	2.041–5.635
Neonatal birth weight	0.000	3.345	0.067	1.000	0.999- 1.000
TC	0.411	2.207	0.137	1.509	0.877–2.597
ApoB	-0.114	0.157	0.692	0.892	0.506–1.571
D-dimer	1.088	11.208	0.001	2.969	1.570–5.614
LDL	1.346	28.965	0.000	3.842	2.353–6.273
WBC	0.757	12.389	0.000	2.132	1.399–3.250

Abbreviations: *TC* Total cholesterol, *ApoB* Apolipoprotein B, *LDL* Low-density lipoprotein,

*WBC* white blood cell

**Table 7:** Multivariable Analysis after stratification by RCOG risk scores

RCOG score	Variable	*B*	Wald	P	OR	95% CI
low-score	mode of delivery	1.223	14.498	0.000	3.399	1.811-6.381
	D-dimer	1.546	3.991	0.046	4.692	1.030-21.380
	LDL	1.641	15.295	0.000	5.160	2.267-11.744
high-score	D-dimer	1.114	9.618	0.002	3.046	1.507-6.158
	LDL	1.434	26.326	0.000	4.194	2.425-7.253
	WBC	1.037	14.918	0.000	2.819	1.666-4.771

Abbreviations: *RCOG* The Royal College of Obstetricians and Gynecologists, *LDL* Low-density lipoprotein,

*WBC* white blood cell

## Discussion

Pregnancy-related venous thromboembolism (VTE) is a leading cause of maternal mortality and morbidity in developed countries [19]. The risk for VTE is highest during the postpartum period. At present, almost all guidelines discriminated between low- and high-risk women, and low-molecular-weight heparin (LMWH) is considered to be the preferred anticoagulant for both prophylaxis and treatment of VTE during pregnancy since it does not cross the placenta and is associated with a lower rate of bleeding complications than that of the unfractionated heparin [20–22]. However, the best strategy to assess and prevent postpartum VTE has not been determined so far [23, 24]. The previous study indicated the high discrepancy between guidelines regarding the prevention of postpartum VTE [25]. The RCOG risk assessment model is one of the most widely used scales for pregnancy-related VTE, but its applicability to the Chinese population is still poorly understood.

Although the mean RCOG score of women in the VTE group was higher than that of the non-VTE group in our study, both groups scored below 2. Furthermore, we found that there was no difference in the incidence of VTE between the high-score and low-score groups stratified according to RCOG ARM. In the high-score group, low-molecular heparin was used for prophylaxis according to the RCOG guideline, but many people still developed VTE. We suggested that it might be related to the inadequate dose of prophylaxis or the need to use other prophylactic strategies in combination. Although there were no clinical risk factors in the low-score group, a significant proportion experienced VTE. Whether these people should be alerted to VTE and treated with thromboprophylaxis requires further study. Our results indicated that the validity of the RCOG RAM for postpartum VTE in Chinese women was not high, but contrary to a previous study which may be related to the relatively small sample size of their study [26]. It reminded us that we still need to look for other risk factors that can be used to effectively assess the risk of VTE. If the risk of postpartum VTE is assessed only based on RCOG guidelines, the administration of low-molecular-weight heparin (LMWH) may be inaccurate and the truly high-risk groups will be ignored and not receive precautions.

Cesarean section was significantly associated with postpartum VTE in the low-risk group based on RCOG RAM in this study. It was speculated that the value of cesarean delivery in predicting postpartum VTE was underestimated in the RCOG model. Women undergoing cesarean section need more individualized thrombosis prevention measures [27], such as the placement of pneumatic compression devices before cesarean delivery for all women according to the American College of Obstetricians and Gynecologists (ACOG) recommendations [14].

In our cohort, we found that some biomarkers could be considered as risk factors for postpartum VTE, including LDL, D-Dimer, and WBC. The ROC curve analysis revealed the best cut-off point for LDL, D-dimer, and WBC count within postpartum 48 h was 2.70 mmol/L, 3.04 mg/L, and 8.64*10^9/L. After stratification by RCOG RAM, the multivariable logistic regression analysis and the ROC analysis further confirmed that RCOG RAM combined with these biomarkers had good discriminative validity, and this model could more accurately predict women with high risks of VTE.

D-dimer is a sensitive marker for VTE and it excludes VTE without the need for further testing among patients with a low clinical probability of PE [28, 29]. However, for pregnant women, D-dimer concentration increased progressively during the pregnancy and peaked on the first postpartum day [30]. It is necessary to find a specific D-dimer threshold to assist the diagnosis. Some studies proved the predictive value of the D-dimer test for pregnancy-related VTE by raising the cutoff value or finding a higher D-dimer reference range [30–32]. Our finding was consistent with previous studies and the recommended cut-off value of D-dimer was 3.04 mg/L.

The low-density lipoprotein (LDL) belongs to lipids. A case-control study indicated that VTE was associated with extreme values of TC and LDL-c [33]. However, van Schouwenburg et al. showed that LDL levels did not influence the risk of venous thromboembolism in a population-based cohort [34]. The association of LDL with postpartum VTE was confirmed for the first time in our study. The LDL ≥ 2.70mmol/L was considered a high-risk factor for postpartum VTE.

It is well known that white blood cells (WBC) are involved in the inflammatory response. Previous research concluded that the inflammatory response may be both the cause and consequence of venous thromboembolism (VTE) [35, 36]. Khorana et al. found that WBC counts more than 11 × 10(9)/L was a predictive variable for hemotherapy-associated VTE [37]. Despite thromboprophylaxis, an elevated WBC count has been suggested as an independent risk factor for in-hospital VTE [38]. Our findings suggested that WBC counts more than 8.64*10^9/L increased the risk of postpartum VTE. Therefore, we could postulate the elevated WBC count involved in the inflammatory response and contributed to the development of postpartum VTE.

The post-hoc analysis also shares the limitations of most retrospective studies, such as biases of an unknown nature and failure to accurately ascertain outcomes. We explored the ability of the RCOG RAM to assess the risk of postpartum VTE in a population with imaging evidence., considering that VTE cannot be confirmed without imaging data. However, the majority of pregnant women delivering in our hospital who were asymptomatic and hence did not have imaging are not included. The majority of this group would fall into the low-score category if assessed by the RCOG RAM and did not go on to have post-partum VTE. It would therefore be useful to know what proportions of this group of asymptomatic pregnant women would be categorised as low or high score according to the RCOG RAM. This study did not include women who used anticoagulants or antiplatelet drugs prenatally because previous studies have shown that the use of these drugs can have some effects on the coagulation indicators, such as D-dimer [39–41], platelets [42–44]. This might lead to a lower risk score and incidence of VTE in the high-score group. The cut-off value of various biomarkers obtained on the ROC curve with an AUC close to 0.5 lacked some reliability. This was a limitation, but it is still meaningful in terms of the final results. In addition, this study is a single-center retrospective study even with a relatively robust sample size, and the generalizability still has to be studied.

## Conclusion

This study had at least two important findings. Firstly, this study indicated that the application of the RCOG RAM to assess the risk of postpartum VTE in China was not the best strategy, while the role of cesarean section in postpartum VTE needs more attention. Secondly, we arrived at the outcome that the LDL, D-dimer, and WBC were potential risk factors for postpartum VTE. Combining these biomarkers (including LDL, D-dimer, and WBC) with RCOG models, predicting VTE outcomes yielded meaningful improvements in the Chinese population.

## Electronic supplementary material

Below is the link to the electronic supplementary material.

**Supplementary Table 1**: The RCOG risk assessment model for VTE

**Supplementary Table 2**: TRIPOD checklist

## Data Availability

We are unable to share our data due to patient privacy. But the date is available from the corresponding author on reasonable requests.

## Abbreviations

VTE: Venous thromboembolism
RCOG: Royal College of Obstetricians and Gynecologists
ARM: Risk assessment model
ROC: Receiver operating characteristic curve
AUC: Area under the curve
TC: Total cholesterol
LDL: Low-density lipoprotein
WBC: White blood cell
BMI: Body mass index
BNP: Brain natriuretic peptide
Hcy: Homocysteine
TG: Total glyceride
HDL: High-density lipoprotein
ApoA1: Apolipoprotein A1
ApoB: Apolipoprotein B
HCT: Red blood cell-specific volume
DM: Diabetes mellitus
GDM: Gestational diabetes mellitus
IVF: In-vitro fertilization
ACOG: American College of Obstetricians and Gynecologists
LMWH: Low-molecular-weight heparin

## References

[1] Gerhardt A, Scharf RE, Beckmann MW, Struve S, Bender HG, Pillny M, Sandmann W, Zotz RB (2000). Prothrombin and factor V mutations in women with a history of thrombosis during pregnancy and the puerperium. N Engl J Med.

[2] Greer IA (2015). CLINICAL PRACTICE. Pregnancy complicated by venous thrombosis. N Engl J Med.

[3] Bourjeily G, Paidas M, Khalil H, Rosene-Montella K, Rodger M (2010). Pulmonary embolism in pregnancy. Lancet.

[4] Pomp ER, Lenselink AM, Rosendaal FR, Doggen CJ (2008). Pregnancy, the postpartum period and prothrombotic defects: risk of venous thrombosis in the MEGA study. J Thromb Haemost.

[5] Royal College of Obstetricians and Gynaecologists. Reducing the risk of venous thromboembolism during pregnancy and the Puerperium: Green-top Guideline No. 37a. 2015.

[6] Jackson E, Curtis KM, Gaffield ME (2011). Risk of venous thromboembolism during the postpartum period: a systematic review. Obstet Gynecol.

[7] O’Shaughnessy F, Donnelly JC, Bennett K, Damkier P, Ainle FN, Cleary BJ (2019). Prevalence of postpartum venous thromboembolism risk factors in an irish urban obstetric population. J Thromb Haemost.

[8] James AH (2009). Pregnancy-associated thrombosis. Hematol Am Soc Hematol Educ Program.

[9] Kalaitzopoulos DR, Panagopoulos A, Samant S, Ghalib N, Kadillari J, Daniilidis A, Samartzis N, Makadia J, Palaiodimos L (2022). Kokkinidis D G and Spyrou N. Management of venous thromboembolism in pregnancy. Thromb Res.

[10] Soma-Pillay P, Nelson-Piercy C, Tolppanen H, Mebazaa A (2016). Physiological changes in pregnancy. Cardiovasc J Afr.

[11] Abdul Sultan A, Grainge MJ, West J, Fleming KM, Nelson-Piercy C, Tata LJ (2014). Impact of risk factors on the timing of first postpartum venous thromboembolism: a population-based cohort study from England. Blood.

[12] James AH, Jamison MG, Brancazio LR, Myers ER (2006). Venous thromboembolism during pregnancy and the postpartum period: incidence, risk factors, and mortality. Am J Obstet Gynecol.

[13] Jacobsen AF, Skjeldestad FE, Sandset PM (2008). Incidence and risk patterns of venous thromboembolism in pregnancy and puerperium–a register-based case-control study. Am J Obstet Gynecol.

[14] American College of O and Gynecologists’ Committee (2018). ACOG Practice Bulletin No. 196: thromboembolism in pregnancy. Obstet Gynecol.

[15] Bates SM, Greer IA, Middeldorp S, Veenstra DL, Prabulos AM, Vandvik PO (2012). VTE, thrombophilia, antithrombotic therapy, and pregnancy: antithrombotic therapy and Prevention of thrombosis, 9th ed: american college of chest Physicians evidence-based clinical practice guidelines. Chest.

[16] Pandor A, Daru J, Hunt BJ, Rooney G, Hamilton J, Clowes M, Goodacre S, Nelson-Piercy C, Davis S (2022). Risk assessment models for venous thromboembolism in pregnancy and in the puerperium: a systematic review. BMJ Open.

[17] Tritschler T, Kraaijpoel N, Le Gal G, Wells PS (2018). Venous thromboembolism: advances in diagnosis and treatment. JAMA.

[18] Hu W, Xu D, Li J, Chen C, Chen Y, Xi F, Zhou F, Guo X, Zhao B, Luo Q (2020). The predictive value of D-dimer test for venous thromboembolism during puerperium in women age 35 or older: a prospective cohort study. Thromb J.

[19] Say L, Chou D, Gemmill A, Tuncalp O, Moller AB, Daniels J, Gulmezoglu AM, Temmerman M, Alkema L (2014). Global causes of maternal death: a WHO systematic analysis. Lancet Glob Health.

[20] Muhamad N, Abu MA, Kalok AH, Shafiee MN, Shah SA, Ismail NAM (2022). Safety and effectiveness of fondaparinux as a postpartum thromboprophylaxis during puerperium among muslim women: a single centre prospective study. Front Pharmacol.

[21] American College of Obstetricians and Gynecologists’ Committee. Thromboembolism in pregnancy; ACOG PRACTICE BULLETIN;number 123. Obstet Gynecol. 2011;e1–e17. 10.1097/AOG.0000000000002706.

[22] Brenner B, Arya R, Beyer-Westendorf J, Douketis J, Hull R, Elalamy I, Imberti D, Zhai Z (2019). Evaluation of unmet clinical needs in prophylaxis and treatment of venous thromboembolism in at-risk patient groups: pregnancy, elderly and obese patients. Thromb J.

[23] Kotaska A (2018). Postpartum venous thromboembolism prophylaxis may cause more harm than benefit: a critical analysis of international guidelines through an evidence-based lens. BJOG.

[24] Bain E, Wilson A, Tooher R, Gates S, Davis LJ, Middleton P (2014). Prophylaxis for venous thromboembolic disease in pregnancy and the early postnatal period. Cochrane Database Syst Rev.

[25] Gassmann N, Viviano M, Righini M, Fontana P, Martinez de Tejada B, Blondon M (2021). Estimating the risk thresholds used by guidelines to recommend postpartum thromboprophylaxis. J Thromb Haemost.

[26] Ge YZ, Zhang C, Cai YQ, Huang HF (2021). Application of the RCOG Risk Assessment Model for evaluating Postpartum venous thromboembolism in Chinese Women: a case-control study. Med Sci Monit.

[27] Lok WY, Kong CW, To (2019). W W K. A local risk score model for venous thromboembolism prophylaxis for caesarean section in chinese women and comparison with international guidelines. Taiwan J Obstet Gynecol.

[28] Bates SM, Jaeschke R, Stevens SM, Goodacre S, Wells PS, Stevenson MD, Kearon C, Schunemann HJ, Crowther M, Pauker SG, Makdissi R, Guyatt GH (2012). Diagnosis of DVT: antithrombotic therapy and Prevention of thrombosis, 9th ed: american college of chest Physicians evidence-based clinical practice guidelines. Chest.

[29] The National Institute for Health and Care Excellence. Guidelines: Venous thromboembolic diseases: diagnosis, management and thrombophilia testing.2020.

[30] Johnson ED, Schell JC, Rodgers GM (2019). The D-dimer assay. Am J Hematol.

[31] Wang M, Lu S, Li S, Shen F (2013). Reference intervals of D-dimer during the pregnancy and puerperium period on the STA-R evolution coagulation analyzer. Clin Chim Acta.

[32] Kovac M, Mikovic Z, Rakicevic L, Srzentic S, Mandic V, Djordjevic V, Radojkovic D, Elezovic I (2010). The use of D-dimer with new cutoff can be useful in diagnosis of venous thromboembolism in pregnancy. Eur J Obstet Gynecol Reprod Biol.

[33] Gonzalez-Ordonez AJ, Fernandez-Carreira JM, Fernandez-Alvarez CR, Venta Obaya R, Macias-Robles MD (2003). Gonzalez-Franco A and Arias Garcia M A. The concentrations of soluble vascular cell adhesion molecule-1 and lipids are independently associated with venous thromboembolism. Haematologica.

[34] van Schouwenburg IM, Mahmoodi BK, Gansevoort RT, Muntinghe FL, Dullaart RP, Kluin-Nelemans HC, Veeger NJ, Meijer K (2012). Lipid levels do not influence the risk of venous thromboembolism. Results of a population-based cohort study. Thromb Haemost.

[35] Galeano-Valle F, Ordieres-Ortega L, Oblitas CM, Del-Toro-Cervera J, Alvarez-Sala-Walther L, Demelo-Rodriguez P. Inflammatory biomarkers in the short-term prognosis of venous thromboembolism: a narrative review. Int J Mol Sci. 2021;22. 10.3390/ijms22052627. 10.3390/ijms22052627 PMC7961591 33807848

[36] Saghazadeh A, Hafizi S, Rezaei N (2015). Inflammation in venous thromboembolism: cause or consequence?. Int Immunopharmacol.

[37] Khorana AA, Kuderer NM, Culakova E, Lyman GH, Francis CW (2008). Development and validation of a predictive model for chemotherapy-associated thrombosis. Blood.

[38] Wang TF, Wong CA, Milligan PE, Thoelke MS, Woeltje KF, Gage (2014). B F. Risk factors for inpatient venous thromboembolism despite thromboprophylaxis. Thromb Res.

[39] Hoke M, Kyrle PA, Philipp K, Pabinger I, Kaider A, Schonauer V, Quehenberger P, Eichinger S (2004). Prospective evaluation of coagulation activation in pregnant women receiving low molecular weight heparin. Thromb Haemost.

[40] Hodo M, Synne GF, Camilla TJ, Mazdak T, Lamya G, Waleed G. The effect of rivaroxaban on the diagnostic value of D-dimer in patients with suspected deep vein thrombosis. Thromb Res 2022 Aug;216:22–4. doi: 10.1016/j.thromres.2022.05.017. 10.1016/j.thromres.2022.05.017 35687980

[41] Nisio MD, Klerk CPW, Meijers JCM, and H R Büller. The prognostic value of the D-dimer test in cancer patients treated withwithout low-molecular-weight heparin. J Thromb Haemost 2005 Jul;3(7):1531–3. doi: 10.1111/j.1538-7836.2005.01413.x. 10.1111/j.1538-7836.2005.01413.x 15978112

[42] Roth 1 GJ, Calverley DC. Aspirin, platelets, and thrombosis: theory and practice. Blood 1994 Feb 15;83(4):885–98. 8111062

[43] Jessica LM, Tabassome. S.Pharmacology of antithrombotic drugs: an assessment of oral antiplatelet and anticoagulant treatments. Lancet. 2015 Jul 18;386(9990):281 – 91. doi: 10.1016/S0140-6736(15)60243-4. Epub 2015 Mar 14. 10.1016/S0140-6736(15)60243-4 25777662

[45] Cines DB, Kaywin P, Bina M, Tomaski A, Schreiber AD. Heparin-associated thrombocytopenia. N Engl J Med. 1980 Oct 2;303(14):788 – 95. doi: 10.1056/NEJM198010023031404. 10.1056/NEJM198010023031404 7412786

