# Supplementary material for: Use of the RCOG risk assessment model and biomarkers to evaluate the risk of postpartum venous thromboembolism
Source: Thromb J. 2023 Jun 12;21:66. doi: 10.1186/s12959-023-00510-6 (PMC10259017; doi:10.1186/s12959-023-00510-6)
Supplement: Supplementary file 1 — Supplementary Table 1: The RCOG risk assessment model for VTE [file 12959_2023_510_MOESM1_ESM.docx]

**Supplementary Table 1: The RCOG risk assessment model for VTE**

| **Risk factors for VTE** | **Score**[**^*^**](https://www.ncbi.nlm.nih.gov/pmc/articles/PMC8274362/table/t6-medscimonit-27-e929904/?report=objectonly#tfn11-medscimonit-27-e929904) |
| --- | --- |
| **Pre-existing risk factors** | |
| Previous VTE (except a single event related to major surgery) | 4 |
| Previous VTE provoked by major surgery | 3 |
| Known high-risk thrombophilia | 3 |
| Medical comorbidities (e.g. cancer, heart failure; active systemic lupus erythematosus, Inflammatory polyarthropathy or inflammatory bowel disease; nephrotic syndrome; type I diabetes mellitus with nephropathy; sickle cell disease; current intravenous drug user) | 3 |
| Family history of unprovoked or estrogen-related VTE in first-degree relative | 1 |
| Known low-risk thrombophilia (no VTE) | 1 |
| Age (>35 years) | 1 |
| Obesity |  |
| BMI ≥30 | 1 |
| BMI ≥40 | 2 |
| Parity ≥3 | 1 |
| Smoker | 1 |
| Gross varicose veins | 1 |
| **Obstetric risk factors** | |
| Pre-eclampsia in current pregnancy | 1 |
| ART/IVF (antenatal only)[^**^](https://www.ncbi.nlm.nih.gov/pmc/articles/PMC8274362/table/t6-medscimonit-27-e929904/?report=objectonly#tfn12-medscimonit-27-e929904) | 1 |
| Multiple pregnancy | 1 |
| Cesarean section in labor | 2 |
| Elective cesarean section | 1 |
| Mid-cavity or rotational operative delivery | 1 |
| Prolonged labor (>24 hours) | 1 |
| PPH (>1 litre or transfusion) | 1 |
| Preterm birth <37^+0^ weeks in current pregnancy | 1 |
| Stillbirth in current pregnancy | 1 |
| Transient risk factors |  |
| Any surgical procedure in pregnancy or puerperium except immediate repair of the perineum, e.g. appendicectomy, postpartum sterilisation | 3 |
| Hyperemesis | 3 |
| OHSS (first trimester only)[^**^](https://www.ncbi.nlm.nih.gov/pmc/articles/PMC8274362/table/t6-medscimonit-27-e929904/?report=objectonly#tfn12-medscimonit-27-e929904) | 4 |
| Current systemic infection | 1 |
| Immobility, dehydration | 1 |

Abbreviation: *RCOG* The Royal College of Obstetricians and Gynecologists, *VTE* venous thromboembolism, *BMI* body mass index, *ART* assisted reproductive technology, *IVF* in vitro fertilization, *PPH* postpartum hemorrhage, *OHSS* ovarian hyperstimulation syndrome
